# Effectiveness, benefit harm and cost effectiveness of colorectal cancer screening in Austria

**DOI:** 10.1186/s12876-019-1121-y

**Published:** 2019-12-05

**Authors:** Beate Jahn, Gaby Sroczynski, Marvin Bundo, Nikolai Mühlberger, Sibylle Puntscher, Jovan Todorovic, Ursula Rochau, Willi Oberaigner, Hendrik Koffijberg, Timo Fischer, Irmgard Schiller-Fruehwirth, Dietmar Öfner, Friedrich Renner, Michael Jonas, Monika Hackl, Monika Ferlitsch, Uwe Siebert, on behalf of the Austrian Colorectal Cancer Screening Model Group

**Affiliations:** 1 Institute of Public Health, Medical Decision Making and Health Technology Assessment; Department of Public Health, Health Services Research and Health Technology Assessment, UMIT - University for Health Sciences, Medical Informatics and Technology, Eduard-Wallnoefer-Zentrum 1, A-6060 Hall in Tirol, Austria; 2 Health Technology and Services Research, University of Twente, Enschede, The Netherlands; 3 Main Association of Austrian Social Security Institutions, Vienna, Austria; 4 Department of Visceral, Transplant and Thoracic Surgery, Medical University of Innsbruck, Innsbruck, Austria; 5 Faculty of Medicine, Johannes Kepler University Linz, Linz, Austria; 6 Medical Association of Vorarlberg, Dornbirn, Austria; 7 Statistics Austrias, Vienna, Austria; 8 Department of Internal Medicine III; Division of Gastroenterology and Hepatology, Medical University of Vienna, Vienna, Austria; 9 Quality Assurance Working Group of Austrian Society of Gastroenterology and Hepatology, Vienna, Austria; 10 Division of Health Technology Assessment and Bioinformatics, ONCOTYROL - Center for Personalized Cancer Medicine, Innsbruck, Austria; 11 Center for Health Decision Science; Department of Health Policy and Management, Harvard T.H. Chan School of Public Health, Boston, MA USA; 12 Institute for Technology Assessment and Department of Radiology, Massachusetts General Hospital; Harvard Medical School, Boston, MA USA

**Keywords:** Colorectal cancer, Screening, State-transition cohort model, Markov model, Colonoscopy

## Abstract

**Background:**

Clear evidence on the benefit-harm balance and cost effectiveness of population-based screening for colorectal cancer (CRC) is missing. We aim to systematically evaluate the long-term effectiveness, harms and cost effectiveness of different organized CRC screening strategies in Austria.

**Methods:**

A decision-analytic cohort simulation model for colorectal adenoma and cancer with a lifelong time horizon was developed, calibrated to the Austrian epidemiological setting and validated against observed data. We compared four strategies: 1) No Screening, 2) FIT: annual immunochemical fecal occult blood test age 40–75 years, 3) gFOBT: annual guaiac-based fecal occult blood test age 40–75 years, and 4) COL: 10-yearly colonoscopy age 50–70 years. Predicted outcomes included: benefits expressed as life-years gained [LYG], CRC-related deaths avoided and CRC cases avoided; harms as additional complications due to colonoscopy (physical harm) and positive test results (psychological harm); and lifetime costs. Tradeoffs were expressed as incremental harm-benefit ratios (IHBR, incremental positive test results per LYG) and incremental cost-effectiveness ratios [ICER]. The perspective of the Austrian public health care system was adopted. Comprehensive sensitivity analyses were performed to assess uncertainty.

**Results:**

The most effective strategies were FIT and COL. gFOBT was less effective and more costly than FIT. Moving from COL to FIT results in an incremental unintended psychological harm of 16 additional positive test results to gain one life-year. COL was cost saving compared to No Screening. Moving from COL to FIT has an ICER of 15,000 EUR per LYG.

**Conclusions:**

Organized CRC-screening with annual FIT or 10-yearly colonoscopy is most effective. The choice between these two options depends on the individual preferences and benefit-harm tradeoffs of screening candidates.

## Background

Colorectal carcinoma (CRC) is the third most common carcinoma and has one of the highest mortality rates worldwide. Most of CRC cases originate from a benign neoplasm (adenoma) [1, 2]. Early detection and removal of these precancerous lesions leads to a significant reduction in CRC incidence and mortality [3].

The chance of early detection increases with CRC screening. Currently, two categories of screening technologies are used: 1) tests for detecting blood, exfoliated DNA or specific enzymes in stool samples and 2) structural exams, including sigmoidoscopy (FSIG), colonoscopy, double-contrast barium enema (DCBE), and computed tomographic colonography (CTC). Although invasive, the structural exams have the advantage that suspicious lesions (adenomatous polyps) can be detected and removed (polypectomy) during the test [4]. However, there are also potential side effects associated with colonoscopy including colonic perforation and major bleeding [5]. Independent of the applied technology, false positive test results and overdiagnosis (i.e., cancers detected at screening that would not have become clinically manifest during one’s lifetime) can lead to discomfort, overtreatment and associated physical and psychological harm. The consequences of diagnostic and therapeutic procedures can also generate stress and anxiety in patients [4, 6, 7].

The Advisory Committee on Cancer Prevention in the European Union recommends that persons 50–74 years old should be screened with guaiac-fecal occult blood test (gFOBT) every 1–2 years. In case of a positive test, colonoscopy should follow [8]. A systematic review on international screening programs showed that for organized screening programs either fecal immunochemical test (FIT) or gFOBT are being used for the initial test due to the higher acceptance of these test technologies [9].

Austria is among those countries in the European Union (EU) with a opportunistic screening program that recommends colonoscopy at intervals of 10 years and annual or biennial gFOBT as an alternative screening strategy [10, 11]. Currently, no organized screening program for colorectal cancer exists in Austria.

As there are currently no head-to-head trials demonstrating that any of the screening strategies is more effective than the others [12], modeling studies have been used worldwide to compare the long-term effectiveness and cost effectiveness of these strategies [13–16]. Cost-effectiveness studies show that CRC screening is cost effective and even cost saving compared to No Screening, however study results differ on which strategy is cost effective [17–20]. Recently, the US Preventive Services Task Force (USPSTF) used three independently created and well-established models (MISCAN, CRC-SPIN, SimCRC) to evaluate benefits, burden (colonoscopies), and harms (colonoscopy complications) of CRC screening strategies [14, 21]. The Task Force estimated that “assuming 100% adherence, the strategies of colonoscopy every 10 years, annual FIT, sigmoidoscopy every 10 years with annual FIT, and CTC every 5 years performed from ages 50 through 75 years provided similar life-years gained (LYG) and a comparable balance of benefit and screening burden” [14].

This study commissioned by the Main Association of Austrian Social Security Institutions aims to systematically evaluate the long-term benefits, harms, costs, benefit-harm and cost-effectiveness relations of different organized CRC screening strategies compared to no screening for average-risk women and men aged 40–75 years in Austria.

## Methods

A decision-analytic Markov state-transition cohort model [22] was developed. The simulation starts with a hypothetical healthy cohort of the general population with average CRC risk. Starting at the age of 20 years, individuals are at age-specific risk for developing one or more adenomas. The evaluation of the screening strategies and calculation of model outcomes start at the age when the decision about the screening program is made (age 40) and are performed lifelong.

The modeling study was performed following international guidelines [23–27]. An Austrian expert panel was established to provide clinical guidance.

### Model design and assumptions

A state-transition Markov model was chosen because it reflects the course of disease of colorectal cancer, with a natural history and disease progression that follows several well-defined histologic and clinical “health states” (Markov states) with transition and event probabilities [23]. The decision-analytic model was programmed and validated using the decision-analytic software package TreeAge Pro 2017 (TreeAge Software Inc., Williamstown, MA, USA).

Within the evaluation of the screening program, repeated screening events are required and time to event is important (e.g., disease progression). As the number of health states is manageable, the model was designed to be analyzed as a cohort simulation [23].

**Fig. 1:**
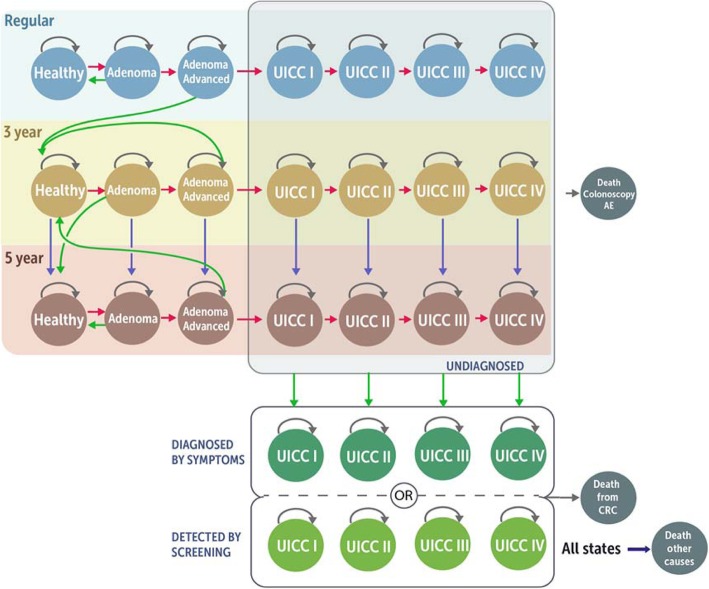
Natural history, impact of screening, and surveillance of the CRC state-transition cohort model. Green arrows – detected, red arrows – progression, blue arrows – switching strategy if adenoma, advanced adenoma or cancer remain undetected or low risk adenoma are detected. UICC - Union for International Cancer Control classification, CRC - colorectal cancer. Regular: regular screening, 3 year: 3-yearly surveillance, 5 year: 5-yearly surveillance. Each bubble represents a health state. Each arrow represents possible transitions between health states, which may occur each year. All individuals start in the healthy state with regular screening. Over time, individuals can develop adenomas. Adenomas can be detected by screening and removed. As a consequence, individuals move back to the healthy state. If advanced adenomas are detected and removed, individuals move back to the healthy state, but with 3-yearly surveillance. If adenomas are not detected, they can progress to advanced adenomas and cancer. Any cancer may be diagnosed at any stage by symptoms or screening. Individuals with diagnosed cancer (symptoms or screening) move to the diagnosed health states where they receive treatment. Individuals with diagnosed CRC may die from CRC. Individuals in any health state may die from other causes according to the age- and sex-specific mortality in Austria. The blue area includes the health states for individuals participating in the regular screening program (according to the investigated screening strategy). The yellow area includes the health states for individuals participating in 3-yearly surveillance (after detection of an advanced adenoma). The brown area includes the health states of the 5-yearly-surveillance program (after detecting non-advanced or no adenoma in the 3-yearly surveillance screening). The health states in these paths are similar compared to the health states of individuals participating in the regular screening program. Only the intervals of screening are shorter compared to the regular screening. If non-advanced adenomas are detected in the regular screening (i.e., according to the screening strategy), individuals will continue with screening using colonoscopy independent from the originally evaluated screening test. Individuals with diagnosed CRC may die from CRC

The model structure including natural history and the impact of screening and surveillance is displayed in Fig. 1. The natural history, that is, occurrence and growth of adenoma and progression to cancer, is modeled starting with healthy individuals at average risk of CRC that enter the model and may develop adenomas. Adenomas may progress to advanced adenoma. Advanced adenomas are defined as “adenoma with villous histology or high-grade dysplasia or ≥ 10mm in size” [28]. Advanced adenomas may further progress and become malignant. Preclinical (i.e., undiagnosed) cancers may progress from stage I to stage IV according to the Union for International Cancer Control (UICC) classification. Cancer at any stage may be diagnosed by symptoms or screening. Adenomas are assumed to be detectable only by screening.

Individuals diagnosed with cancer are assumed to be treated according to the Austrian clinical guidelines [11] reflected in the Austrian claims data of the Main Association of Austrian Social Security Institutions. According to the structural assumption of the model, individuals technically remain in the health state determined after the cancer diagnosis for their remaining lifetime until they die from CRC or other causes. In those“health states (diagnosed cancer states), stage-specific follow-up treatment and survival, which also accounts for further disease progression, are considered.

Evaluated screening strategies may alter the risk of cancer progression and survival probability due to the removal of adenomas before they become malignant or due to early detection (with potential removal) of cancer. Adverse effects from colonoscopy (confirmatory or screening) leading to hospitalization or death are also considered. At any point in time, individuals may die from other causes.

The following model assumptions were made: [1] the model simulates an average number of lesions, meaning that the progression of single adenomas was not simulated [2]; adenomas cannot regress, because regression of adenoma is rare and evidence from literature is limited [21] [3]; age-specific risk for adenoma, and other risk factors such as gender and anatomical adenoma location as well as age-specific adenoma progression were not explicitly modeled [4]; incidental detection of asymptomatic disease was not considered, adenomas can only be detected by screening [5]; symptomatic patients would receive confirmatory colonoscopy and therefore face the risk of adverse events. For confirmatory colonoscopies in symptomatic patients, false negative results were assumed to be negligible for our evaluation.

### Screening population and strategies

The implemented screening strategies include follow-up screening algorithms (surveillance) based on the Austrian guidelines [11] and recommendations of the European Society of Gastrointestinal Endoscopy (ESGE) [28] and were confirmed by the Austrian expert panel. Four screening strategies are considered: 1) No Screening, 2) annual immunochemical fecal occult blood test (FIT) at age 40–75 years, 3) annual guaiac-based fecal occult blood test (gFOBT) at age 40–75 years, and 4) ten-yearly colonoscopy at age 50–70 years. Other index tests were not considers by the experts for several reasons including limited relevance in the Austrian setting (sigmoidoscopy), additional radiation and missing recommendation for routine use (CT colonography) or limited evidence on test accuracy (DNA stool tests).

In the screening strategies with annual FIT and gFOBT, the patients with a positive blood test result undergo diagnostic colonoscopy.

In all strategies, patients with detected CRC are treated according to Austrian treatment guidelines. They continue with follow-up examinations and do not enter the regular screening program again. Identified non-adenomas and advanced adenomas are removed by polypectomy and individuals continue screening according to the assumptions described below.

In the screening strategies with annual fecal occult blood tests, patients with detected non-advanced adenomas continue screening with colonoscopy every 10 years. The detection of advanced adenomas leads to 3-yearly surveillance with colonoscopy.

Similarly, in the colonoscopy screening program individuals continue with the 10-year colonoscopy screening interval, if non-advanced adenomas are detected and patients with detected advanced adenomas, are referred to 3-yearly surveillance.

Patients undergoing 3-yearly surveillance continue surveillance in 3-years intervals only if an advanced adenoma was found in the following surveillance examination. If non-advanced or no adenomas are found, these individuals are referred to 5-yearly surveillance with colonoscopy. They will continue the 5-yearly surveillance as long as no advanced adenomas are detected. A detection of advanced adenomas will lead to 3-yearly surveillance.

In all strategies, surveillance examinations until the age of 75 are considered.

### Natural history data and model calibration

Natural history parameters for the progression of the disease were estimated in three steps. First, epidemiological data (cancer incidence, cancer stage distribution) were determined from Statistics Austria [29] and published literature serving as starting parameter sets and calibration targets. Second, the model was calibrated in a hierarchical fashion using optimization algorithms (Nelder Mead and Basinn-Hoping) and third, a final parameter adjustment was performed to meet the calibration-target distribution for all cancer stages. Further details on model calibration and the natural history parameter values are reported in the Additional file 1.

### Colorectal cancer survival and mortality from other causes

The age-specific mortality rates from other causes were based on Austrian statistical life tables for the year 2016 from Statistics Austria [30]. Mortality rates for age groups over 100 years were extrapolated applying an exponential distribution. CRC-specific mortality (post-diagnosis) was derived from Statistics Austria (2010–2014), extrapolated and adjusted for screenning detection and symptom detection [29]. Hazard ratios between these two modes of detection for different cancer stages were derived from Brenner et al. [31] (see Additional file 1: Table S7 and S8).

### Screening test accuracy

For FIT, sensitivity for advanced adenoma (36.7%), CRC (87.2%) and specificity for both adenoma and CRC (92.8%) was obtained from a meta-analysis (22 studies pooled, 174,469 patients, brand: OC-Sensor) [32]. Differences in the results for the specificity of advanced adenoma (93.4%; 95% CI: 90.2–95.6%) and CRC (92.8%; 95% CI: 90.6–94.5%) were not significant, therefore the specificity for CRC was selected as overall specificity of the test. The sensitivity of FIT for non-advanced adenomas (7.6%) was obtained from a large clinical trial (9989 patients, brands: OC FIT-CHEK, Polymedco) [33].

For gFOBT, sensitivity for CRC (72.2%) and specificity (90.0%) was obtained from a meta-analysis (6 studies pooled, 7564 patients, brands: Hemoccult, Hemoccult II, Hemoccult Sensa) [34]. Reported sensitivity for CRC for the proximal (62.6%) and distal colon (75.4%) was pooled according to the distribution of anatomical location (proximal 25%, distal 75%) [35]. Sensitivity for adenomas (9.5%) and advanced adenomas (23.9%) were determined from a modeling study from the USPSTF (brand: Hemoccult Sensa) [36]. Sensitivity for adenomas was reported in the USPSTF-study only by adenoma size (1-5 mm 7.5%; 6-9 mm 12.4%; > 10 mm 23.9%) therefore, a pooled sensitivity for adenomas 1–9 mm (1-5 mm 60.3%, 6-9 mm 39.7% [35]) was calculated and for advanced adenomas the sensitivity for adenomas > 10 mm was considered.

For colonoscopy, a meta-analysis was conducted due to missing pooled data. As a result, sensitivities of colonoscopy for non-advanced adenomas was 69.0% and for advanced adenomas 86.7% per patient [37]. The sensitivity of colonoscopy for CRC (94.7%) was obtained from a published meta-analysis including trials where computed tomographic colonoscopy was compared to optical colonoscopy (49 studies; 11,151 patients) [38]. The specificity of colonoscopy for adenomas and for CRC was assumed to be 100% according to the Austrian expert panel.

Furthermore, it was assumed that the test accuracy of confirmatory colonoscopy after a positive fecal blood test result is independent of the first fecal blood test result. Potential changes of the sensitivity and specificity in a long series of consecutive fecal occult blood tests due to specific characteristics of lesions were not considered due to a lack of information. Information on test accuracy parameter values is summarized in the Additional file 1: Table S2.

### Costs

Direct medical costs were derived from the perspective of the Austrian public-health care system. Both medical outpatient- and inpatient-care costs were based on original data from the Main Association of Austrian Social Security Institutions (HVB) and include costs of tests, staging, medication follow-up, screening, treatments of complications and average cost for end-of-life treatment of colorectal cancer and rectal cancer [39]. All costs were inflated to the index year 2017 by using the Consumer Price Index (CPI) for Austria from the OECD [40]. Table 1 presents the aggregated costs taking into account the relative frequency distribution of cancer location, cancer stage and medication options (reported in the Additional file 1) [29]. The cancer locations are classified using the 10th revision of the International Statistical Classification of Diseases and Related Health Problems (ICD-10) and include malignant neoplasms of colon (ICD-10 C18), of rectosigmoid junction (ICD-10 C19) and of rectum (ICD-10 C20) [1]. Further information and cost data are provided in the Additional file 1: Table S3, S4 and S5.

**Table 1:** Aggregated costs of tests, staging, inpatient, medication, follow-up, screening, complications and end-of-life (index year 2017)

Item	Costs at index year 2017, EUR
Costs for tests	
Colonoscopy	228
Polypectomy	64
gFOBT (gFOBT stool test only)	37 (0.83)
FIT (FIT stool test only)	41 (0.89)
Staging costs (weighted mean of colorectal cancer and rectal cancer)	461
Aggregated inpatient-care costs (weighted mean of colorectal cancer and rectal cancer at UICC level)	
UICC I	13,831
UICC II	18,699
Costs UICC III	19,038
Costs UICC IV	24,059
Aggregated medication costs (UICC IV)	12,433
Aggregated follow-up costs (weighted mean of colorectal cancer and rectal cancer separately for follow-up year, including medical consultation, tumor marker laboratory, colonoscopy/rectoscopy, CT)	
Costs: Year 1	552
Costs: Year 2	367
Costs: Year 3	349
Costs: Year 4	419
Costs: Year 5	237
Costs: Year 9, 14, lifelong every 60 months	228
Costs for screening program	
Colonoscopy screening program (10-yearly)	1,950,353
Stool-based screening program (annually)	4,118,142
Costs of complications (inpatient stay)	
Surgical procedures	23,258
Inpatient stay	5250
End-of-life costs (inpatient care + medication)	
One-time costs, cancer death at UICC I and UICC II	55,530
One-time costs, cancer death at UICC III	36,492

*EUR* Euro, *gFOBT* Guaiac-fecal occult blood test, *FIT* Fecal immunochemical test, *UICC* Union for International Cancer

### Model analyses and outcomes

The Markov model has a cycle length of 1 year, simulating individuals until death. Half-cycle correction is used at start and termination of the model.

#### Outcomes

Predicted outcomes are: benefits expressed as life-years gained [LYG], CRC-related deaths avoided and CRC cases avoided; harms expressed as additional complications due to colonoscopy (physical harm) and positive test results (psychological harm); and lifetime costs. Related differences (increments) of these outcomes when compared to the next non-dominated strategy. Benefits and harms are displayed in an population fact box [41]. Tradeoffs were expressed as incremental harm-benefit ratios and incremental cost-effectiveness ratios.

The clinical tradeoffs between benefits and harms for a screening strategy that provides more benefits but also lead to additional harms in comparison to an alternative strategy are expressed as so called incremental harm-benefit ratio (IHBR). The IHBR is calculated by dividing the difference in harms (incremental harm, e.g. additional positive test results, adverse events) by the difference in the chosen measure of benefit (incremental benefit, e.g., additional life-years gained, cancer cases avoided). The IHBR provides information of additional harms individuals will be exposed to gain one unit of benefits in a screening strategy compared to a less beneficial/effective strategy. The primary IHBR of our analysis was defined as additional psychological harm due to positive test results for one additional life-year gained when using one strategy compared to another. Similarly, the secondary IHBR was defined as the psychological harm due to additional positive test results per CRC-related death avoided or per CRC avoided.

Economic outcomes include lifetime costs and discounted incremental cost-effectiveness ratios (ICER) expressed in additional costs (in EUR) per life-year gained (LYG). The ICER is calculated by dividing the discounted incremental costs between two alternatives by the discounted incremental health effects between these two alternatives. An annual discount rate of 3% was applied for the cost-effectiveness analysis. Strategies are considered dominated if they provide less health benefit at higher costs when compared to any other strategy. Therefore, dominated strategies should not be considered by decision makers and no ICER is calculated. Furthermore, extended dominance is applied to eliminate strategies, for which costs and benefits are dominated by a mix of two other alternatives. A dominant strategy provides better health effects at lower cost compared to other strategies [42, 43].

#### Base-case analysis

For the base-case analysis, we chose a sustained strategy comparison, that is, full adherence to screening strategies including follow-up and surveillance tests was assumed to provide a strict comparison of the intended strategies without dilution by non-adherence.

#### Sensitivity analysis

We performed one-way and two-way deterministic sensitivity analyses as well as deterministic scenario analyses on crucial input parameters and assumptions to evaluate the robustness of the results and to identify future research priorities. In the one-way sensitivity analyses, we varied the sensitivity for fecal occult blood tests from 0 to 100% to account for declining sensitivity of consecutive tests because it is likely that sensitivities of repeated tests in the same individual are dependent conditional on disease, and therefore, may be substantially lower in individuals with prior false negative test results. Increasing costs of new therapies were considered by increasing the inpatient-care costs of patients in tumor stage UICC IV by up to 50%. The cost of colonoscopy and polypectomy was increased by up to 100%. The discount rate was varied within the range of 0 to 10%.

In the two-way sensitivity analyses, the sensitivity parameters for fecal occult blood tests and colonoscopy were reduced by up to 50% and increased by up to 10% simultaneously. In a scenario analysis, the cost for screening colonoscopy and polypectomy was assumed to be EUR 352 and EUR 98, respectively. In a second scenario analysis, the participation rates were assumed to be 20.0% for colonoscopy and 38.9% for FIT according to Austrian experiences and 31.1% for gFOBT assuming a 20% lower acceptance rate of gFOBT compared to FIT [44, 45]. Furthermore, the participation rates were assumed to be 28.0% for colonoscopy, 31.1% for gFOBT and 38.9% for FIT. In a two-way sensitivity analysis, the participation rates of colonoscopy and fecal occult blood tests were simultaneously varied from 10 to 100%. Finally, the CRC related mortality rates were assumed to be independent of the mode of detection (by screening or symptoms). Relative cancer stage- specific survival probabilities reported by Statistics Austria 2010–2014 including a mix of screen- and symptom-detected patients were applied for all patients diagnosed with cancer (see Additional file 1: Table S9).

#### Model validation

The model was validated internally and externally on several levels: (1) face validity (i.e., by clinical experts, modeling experts, and patient representatives), (2) internal validation (e.g., debugging, consistency and plausibility checks), (3) external validation with epidemiological data from Statistics Austria [29] (cumulative cancer mortality at age 75) and data from the literature.

## Results

### Validation

The calibrated natural history model predicts a cumulative CRC-related mortality of 1.74% at the age of 75. Statistics Austria reports a cumulative mortality of 1.97% for the years 1995–1999 [29]. The relative difference of − 4.28% is reasonable according to the Austrian expert panel.

### Base-case analysis screening-related benefits and harms

In comparison to No Screening, screening a cohort of 1000 40-year-old individuals is expected to gain 394 LYG with 10-yearly with colonoscopy from age 50 to 70, 480 LYG with annual gFOBT from age 40 to 75, and 491 LYG with annual FIT from age 40 to 75. These and the following results represent total results for the screening strategies including index testing, further diagnostics, surveillance, treatment and follow up interventions. Colonoscopy yielded 30 averted CRC-related deaths, and both FIT and gFOBT yielded 35 averted CRC-related deaths per 1000 screened individuals. In terms of CRC incidence, colonoscopy averted 61, gFOBT 66 and FIT 69 CRC cases per 1000 screened individuals, respectively.

In comparison to no Screening the screening strategies lead to unintended psychological and physical harms. The colonoscopy screening strategy leads to 679 expected positive test results per 1000 individuals. In comparison to colonoscopy, gFOBT results in around four times as many positive test results (*n* = 2797), and FIT to more than three times as many positive test results (*n* = 2206). In all strategies, the additional complications due to colonoscopy leading to hospitalization were very low, at 1–2 expected cases per 1000 screenees. The comparative effectiveness (i.e., benefit outcomes) and unintended harms are summarized in the Additional file 1: Table S10.

The benefits and harms of the non-dominated screening strategies FIT and colonoscopy are displayed in an population fact box (see Table 2) and in an individual fact box (see Table 3) in order to guide decisions of payers, physicians and screening candidates. It must be mentioned that the results in the fact boxes are a consequence of both different screening intervals and different screening tests.

**Table 2:** Comparative population fact box for benefits and harms (per 1000 persons)

Outcome	10-yearly Colonoscopy	Annual FIT	Differences FIT vs. Colonoscopy
Life-years gained:	394	491	97
CRC-related deaths averted	30	35	5
CRC cases averted	61	69	8
Additional complications due to colonoscopy (hospital admissions)	1.2	1.2	0
Total positive test results	679	2206	1527

Numbers pertain to a cohort of 1000 persons 40 years of age who were followed until death in comparison to No Screening. Full adherence to screening strategies including follow-up and surveillance tests was assumed. *CRC* Colorectal cancer, *FIT* Fecal immunochemical test screening strategy. FIT: 40–75 years old average - risk men and women. Colonoscopy: 50–70 years old average - risk men and women, all screening strategies include index testing, further diagnostics (including colonoscopy), surveillance (colonoscopy), treatment and follow up interventions; annual guaiac-fecal occult blood test screening strategy is dominated by annual FIT in benefit - harm analysis and, therefore, not included in table.

**Table 3:** Comparative individual fact box for benefits and harms (per person)

Outcome:	10-yearly Colonoscopy	Annual FIT	Differences FIT vs. Colonoscopy
Life-weeks gained^a^	21	26	5
Probability of dying from CRC (%)	0.8	0.3	− 0.5
Probability of developing CRC (%)	2.2	1.4	−0.8
Mean number of complications due to colonoscopy (hospital admissions)	0.0012	0.0012	0
Mean number of positive test results	0.7	2.2	1.5

Numbers pertain to an individual average 40 years of age who were followed until death. Full adherence to screening strategies including follow-up and surveillance tests was assumed. ^a^in comparison to No Screening, CRC - colorectal cancer, FIT - fecal immunochemical test screening strategy. FIT: 40–75 years old average - risk men and women. Colonoscopy: 50–70 years old average - risk men and women, all screening strategies include index testing, further diagnostics (including colonoscopy), surveillance (colonoscopy), treatment and follow up interventions; annual guaiac-fecal occult blood test screening strategy is dominated by annual FIT in benefit harm analysis and, therefore, not included in table.

In particular, the individual fact box translates population numbers into expected values per one individual, that is, one screening candidate. For example, the individual fact box presented in Table 3 shows that moving from 10-yearly colonoscopy to annual FIT is associated with an average gain of 5 life-weeks at the cost of 1.5 additional positive test results.

In order to gain one life-year with annual FIT compared to 10-yearly colonoscopy, there is an expected incremental unintended psychological harm of additional 16 positive test results (derived from Table 2).

In order to avoid one CRC-related death with annual FIT compared to 10-yearly colonoscopy, there is a psychological harm of more than 300 additional positive test results.

In order to avoid one CRC-case with annual FIT compared to 10-yearly colonoscopy, there is an incremental expected psychological harm of additional 200 positive test results.

### Cost effectiveness

**Fig. 2:**
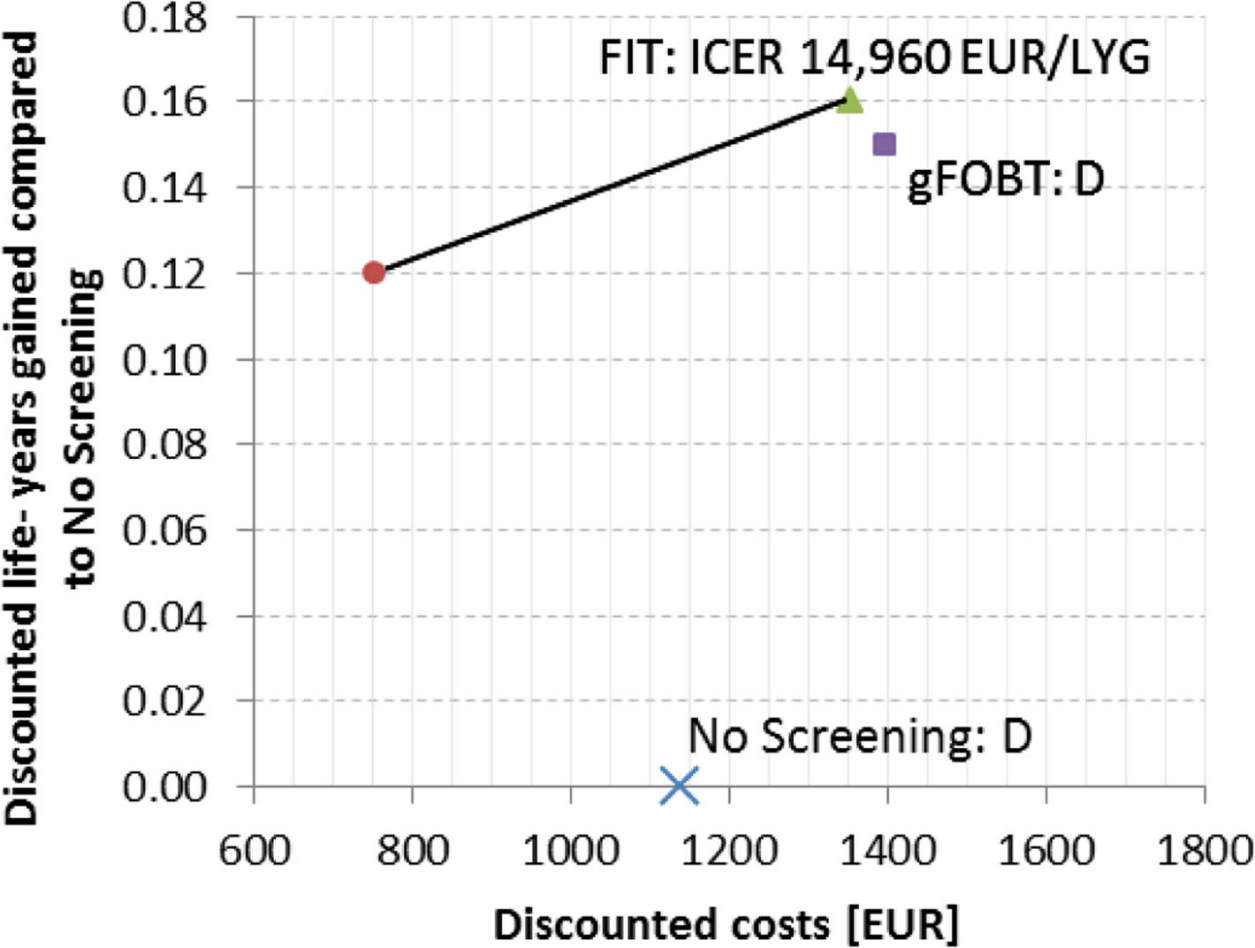
Cost effectiveness of colorectal screening strategies. Blue cross - No Screening, red circle - colonoscopy, purple square - gFOBT, green triangle - FIT. D - dominated, ICER - incremental cost-effectiveness ratio, gFOBT - guaiac-fecal occult blood test screening strategy, FIT - fecal immunochemical test screening strategy, EUR - Euro, LYG - life-years gained, FIT and gFOBT: 40–75 years old average-risk men and women, annual. Colonoscopy: 50–70 years old average-risk men and women, 10-yearly. All screening strategies include index testing, further diagnostics (including colonoscopy), surveillance (colonoscopy), treatment and follow up interventions. Base-case analysis: assumes full participation and adherence

Details of the incremental cost-effectiveness analysis are shown in Table 4 and Fig. 2. Based on our base-case analysis with a screening adherence of 100% in all screening strategies, the strategy No Screening (discounted costs: EUR 1138) and the gFOBT strategy (discounted costs: EUR 1398, LYG in comparison to No Screening 0.15 years) are dominated, and are therefore no efficient choices for decision makers. In 40-year old individuals, colonoscopy leads to an average of 0.12 discounted life-years gained (i.e., 44 life-days gained) when compared to No Screening and to average discounted lifetime costs of EUR 754. In contrast, the FIT strategy leads to an average of 0.16 discounted life-years gained (i.e., 58 life-days gained) when compared to No Screening and to average lifetime costs of EUR 1352. The corresponding ICER of switching from colonoscopy to FIT is EUR 14960/LYG.

**Table 4:** Health economic results of colorectal cancer screening programs

Screening Strategy	LYG	Disc. LYG	Costs [EUR]	Disc. costs [EUR]	Disc. incr. LYG	Disc. incr. Costs [EUR]	ICER [EUR/LYG]
No Screening	–	–	2890	1138	–	–	dominated
10-yearly Colonoscopy	0.39	0.12	1440	754			
Annual gFOBT	0.48	0.15	2341	1398	–	–	dominated
Annual FIT	0.49	0.16	2257	1352	0.04	598	14,960

*LYG* Life-years gained compared to No Screening per individual, *disc* Discounted, *ICER* Incremental cost-effectiveness ratio, *gFOBT* Guaiac-fecal occult blood test screening strategy, *FIT* Fecal immunochemical test screening strategy, *EUR* Euro. FIT and gFOBT: 40–75 years old average - risk men and women. Colonoscopy: 50–70 years old average - risk men and women, all screening strategies include index testing, further diagnostics (including colonoscopy), surveillance (colonoscopy), treatment and follow up interventions. Full adherence to screening strategies including follow-up and surveillance tests was assumed.

### Benefit-harm-cost tradeoffs

If, based on the benefit-harm analysis or based on personal preferences regarding screening burden, the first choice between annual stool blood tests and 10-yearly colonoscopy is the colonoscopy, then the colonoscopy program is considered the best screening option as well as cost saving compared to all other strategies.

If, however, based on the benefit-harm analysis, the first choice between the compared strategies is annual FIT, then the cost-effectiveness depends on the payer’s willingness-to-pay. In this case with a payer’s willingness-to-pay threshold above EUR 15000 per life-year gained, the annual FIT strategy is considered the best as well as a cost-effective screening option.

### Sensitivity analyses

An overview of the results of the one-way sensitivity analyses comparing colonoscopy and FIT are provided in Table 5. Model-predicted base-case cost-effectiveness results were particularly sensitive to sensitivities of fecal occult blood stool tests and colonoscopy, discount rate as well as participation rates. FIT would be more effective and less costly than all other strategies assuming a participation rate of 28% for Colonoscopy, 38.9% for FIT and 31.1% for gFOBT. An increase in costs of inpatient care of patients in cancer stage UICC IV and the application of CRC-specific mortalities unadjusted for the mode of cancer detection (detected by screening or symptoms) showed only minor effects on the ICER.

**Table 5:** Summary one-way sensitivity analyses

Analysis / adapted parameters	Comments	ICER [EUR/LYG] FIT vs. Colonoscopy	
Base case		14,960	
Survival probability	Survival probability for patients diagnosed with cancer unadjusted for mode of detection	17,595	
Participation rate	Colonoscopy 20.0%; FIT 38.9%, gFOBT 31.1%	FIT is dominant	
Participation rate	Colonoscopy 28.0%; FIT 38.9%, gFOBT 31.1%	FIT is dominant	
Costs examination	Cost for screening colonoscopy examination EUR 352, polypectomy EUR 98	15,853	
Test accuracy	Relative reduction of sensitivity of FIT and gFOBT (0%; 60%)^a^	14,960	58,131
Discount rate	Assumed discount rate (0; 10%)	8493	48,911
Costs examination	Relative increase in costs of colonoscopy examination and polypectomy (0, 100%)	14,960	16,156
Costs treatment	Inpatient-care costs treating cancer stage UICC IV (relative increase 0, 50%)	14,960	14,678

^a^further reduction lead to FIT being the dominated by colonoscopy, *gFOBT* Guaiac-fecal occult blood test screening strategy, *FIT* Fecal immunochemical test screening strategy, dominant – screening strategy that is both more effective and less costly compared to all other strategies examined. *ICER* Incremental cost-effectiveness ratio, *EUR* Euro, *LYG* Life-years gained, *UICC* Union for International Cancer Control classification. FIT and gFOBT: 40–75 years old average - risk men and women, annual. Colonoscopy: 50–70 years old average - risk men and women, 10-yearly, all screening strategies include index testing, further diagnostics (including colonoscopy), surveillance (colonoscopy), treatment and follow up interventions

The analysis of reduced sensitivity of repeated fecal occult blood test (i.e., dependence of sensitivity conditional on disease) indicate that an overall 70% reduction would lead to a similar life expectancy for the FIT and the colonoscopy strategy. Such a reduction would imply that colonoscopy becomes a dominant strategy. An overall reduction of 60% sensitivity leads to similar life expectancy of gFOBT and colonoscopy. Additional graphical results for the one-way sensitivity analysis on test sensitivity and the results of the two-ways sensitivity analyses on test accuracies as well as participation rates are presented in the Additional file 1.

## Discussion

Based on our results, colorectal cancer screening with an annual FIT is more effective than all other investigated screening strategies when considering long-term outcomes such as life expectancy, risk of colorectal cancer, and mortality due to colorectal cancer. The annual gFOBT strategy is less effective and was dominated in the economic evaluation. The 10-yearly colonoscopy screening strategy is less effective compared with annual FIT in terms of remaining life expectancy, risk of colorectal cancer, and mortality due to colorectal cancer, but it is also less costly. Moving from colonoscopy to FIT has a discounted incremental cost-effectiveness ratio of EUR 14960/LYG. The benefit-harm analysis, however, shows that in order to gain one life-year with annual FIT compared to 10-yearly colonoscopy, there is an expected incremental unintended psychological harm of additional 16 positive test results. In order to avoid one CRC-related death with annual FIT compared to 10-yearly colonoscopy, there are more than 300 additional positive tests.

Our findings are consistent with the results of other published modeling studies showing that No Screening is clearly dominated [14, 15, 17]. However, in the literature, there is no clear evidence about what is an optimal or cost-effective screening test or strategy [46]. Results differ because of applications in different health care settings, main model assumptions including age of initiation and termination of screening, screening intervals, surveillance, sensitivities of tests (depending on brand, cut-off values and source of information), evaluation period, and country-specific epidemiology as well as country-specific cost structures. As a consequence, a wide variety of screening strategies are being offered worldwide.

The USPTF reported colonoscopy every 10 years and annual FIT to be recommendable strategies in terms of effectiveness [17]. With colonoscopy, slightly more LY could be gained compared to FIT. In our analysis, FIT provides more LY in comparison with colonoscopy. In the USPTF study, no high sensitivity gFOBT strategy was recommanded [14]. To our knowledge, there is no study comparing exactly the same screening scenarios including surveillance follow up based on Austrian guidelines. In the systematic review of Lansdorp-Vogelaar et al., discounted LYG of annual gFOBT in comparison to No Screening ranges between 0.019 and 0.16 and for colonoscopy between 0.019 and 0.18 (studies published year 2000 onwards) [17]. The results of our base-case analysis are within these ranges (gFOBT discounted LYG 0.15, colonoscopy discounted LYG 0.12). In this review, approximately half of the studies found FIT to be dominant and the other half found FIT to be dominated by gFOBT Hemoccult Sensa based on US cost estimates [17]. For a willingness-to-pay of $ 20,000/LYG 10-yearly colonoscopy was predominantly the optimal option. As another example, Zauber evaluated screening strategies in the US initiated at the age of 50 until the age of 80 following the cohort for a maximum age of 100. Reported LYG for a cohort of 1000 individuals are 238 with FIT, 240 with gFOBT (Hemoccult Sensa) and 243 with colonoscopy. Differences in the absolute values in comparison to our study (colonoscopy LYG 394, gFOBT LYG 480, FIT LYG 491) may be caused by different ages of initiation and termination, assumptions about test sensitivities and surveillance [47]. The EUnetHTA report of gFOBT and FIT concluded that FIT should be the preferred choice of those two fecal occult blood test due to several characteristics including higher sensitivity and higher participation rate [48].

A specific strength of our study is that based on the natural history of the disease, we transparently described and systematically evaluated the effect of the sensitivity of different screening tests including surveillance, capturing stage shift and incorporating survival probabilities depending on the mode of detection (screening, symptoms) over a lifelong time horizon. Settings and uncertain variables were assessed systematically in sensitivity analyses to examine the robustness of the model’s predicted results and to identify further research priorities. This is a typical example of a situation where decision-analytic modeling offers a transparent and systematic decision aid and complements the results from randomized clinical trials. Results were presented in systematic fact boxes (Tables 2 and 3) to support communication of multiple benefits and harm outcomes from the public health and individual perspective.

As all decision analyses, our study has several limitations. First, we did not consider shorter screening intervals for colonoscopy or biennial intervals for fecal occult blood tests. The improved clinical benefits of annual fecal occult blood tests in comparison to 10-yearly colonoscopy can be partly explained by the fact that the 10-year sensitivity (Sensitivity_10y_ = 1-(1-Sensitivity_1year_)^10) for FIT and gFOBT is higher than the sensitivity of colonoscopy in advanced adenomas and cancer. In adenomas, the 10-year sensitivity for FIT and gFOBT is only slightly lower than the sensitivity of colonoscopy, which is performed only once every 10 years (see Additional file 1: Table S11). Therefore, shorter screening intervals for colonoscopy should also be investigated.

Second, we assumed that the test accuracies of consecutive annual fecal blood tests are independent conditional on disease. If there is a biological reason why the test failed to detect lesions that do not change over time, this assumption does not hold (e.g., lesions in the right-sided colon are usually non-polypoid or flat, which is assumed to be associated with less bleeding) [34]. This means that undetected lesions associated with less bleeding may in practice decrease overall sensitivity for fecal occult blood tests of certain persons over time. Our results may therefore overestimate the effectiveness of repeated fecal occult blood tests and underestimate costs, because missed adenomas may progress to cancer and may therefore, also lead to further treatment cost. A simplified first sensitivity analysis showed that a reduced sensitivity of FIT by an overall factor of 0.3 would lead to similar remaining life expectancy for FIT and colonoscopy. For a more precise analysis, a microsimulation that allows for modeling separate lesions with the respective location and further characteristics would be required. For a confirmatory colonoscopy, it is more likely that the sensitivity is closer to the sensitivity of a colonoscopy in a patient without a pretest since the sensitivity is less dependent on the prevalence of the disease. In practice, however, a physician examining a patient with a positive stool test may adapt clinical practice, spending more time and, therefore, increasing the chance to detect lesions. With respect to the applied parameter values, test sensitivity and specificity data for primary screening tests were based upon meta-analysis results including data from randomized clinical trials. However, sensitivity and specificity in real-world settings may also be reduced due to clinical practice, which differs from a strictly defined setting of a clinical trial and may depend on physicians’ experiences and learning curves with new technologies etc.

The reported accuracies of fecal occult blood tests are usually calculated assuming standard colonoscopy to be the “gold standard”. Standard colonoscopy, however, is not a perfect test. For an improved approximation of the sensitivities of fecal blood tests, the relative sensitivities provided by published studies should be adjusted by the sensitivities of colonoscopy. These adjusted sensitivities should be applied in future scenario analyses.

Reported sensitivities of gFOBT and FIT vary considerably. Sensitivities of gFOBT for advanced adenomas are reported in a recent systematic review ranging from 31.4–41.3% (median 30.8%) and for CRC ranging from 37.1–79.4% (median 62.9%) [5]. An EUnetHTA report for Austria provides a range of 13–63% for the sensitivity of gFOBT [48]. A meta-analysis on Hemoccult (an outdated test) only reported a sensitivity of 14% for advanced adenomas and sensitivity for CRC of 47.4% [32]. Our assumptions for the sensitivity of advanced adenomas of 23.9% were based on a recent modeling study [14] and sensitivity for CRC (72.2%) was based on a recent meta-analysis [34]. Sensitivities of FIT for advanced adenomas are reported in a recent systematic review ranging from 6 to 44% (median 28%) and for CRC ranging from 25 to 100% (median 88%) [5]. A German study on “immoCARE-C” reported sensitivities depending on cut-off values (37% for polyps > 1 cm cut-off 50, CRC not reported for cut-off 50 and lower) [49]. A recent clinical trial on 9989 patients reported a sensitivity of FIT for advanced adenomas of 23.8 and 73.8% for CRC [33]. Our assumptions on FIT sensitivity (advanced adenoma 36.7%, CRC 87.2%) are based on a recent meta-analysis, for “OC sensor” [32].

Third, the setting of perfect adherence to screening in the base-case analysis including follow-up and surveillance tests provides the maximum achievable benefit for each strategy from the patient perspective (if compliant). Implemented screening programs often face the problem to achieve such benefits and adherence may also be dependent on the test itself, comorbidities, or respective mass campaigns [44, 45, 50, 51]. This is important for a population perspective and public health considerations. Adherence rates were, therefore, adjusted in the sensitivity analysis focusing on adherence to the primary screening test. As a result, assuming a participation rate of 28% for colonoscopy, 39% for FIT and 31% for gFOBT, FIT would become dominant, that is more effective and less costly than all other strategies. More complex adherence patterns that include adherence for confirmatory colonoscopy, for positive fecal occult blood tests or surveillance could be investigated further.

Fourth, we used reimbursement costs for the inpatient care of CRC cases derived from Austrian health insurances. These claims data contain still some level of uncertainty and, in addition, actual costs, for example in hospitals, may be higher. Therefore, our results are rather conservative. The ranking and dominance of strategies should be independent of this fact. In future, treatment costs may not describe the real costs, because promising immunotherapies that enter clinical practice may increase costs substantially. The sensitivity analysis on increased costs for patients in stage UICC IV, however, did not show much impact on the results since No Screening and gFOBT remained dominated and the ICER comparing colonoscopy and FIT decreased slightly in favor of FIT.

Fifth, to define epidemiological calibration target values for the distribution of cancer stages in the Austrian population, patients with reported unknown cancer stages were distributed among all cancer stages assuming random causes and death certificate only cases (DCO) were assumed to be more severe and, therefore, distributed among UICC III and UICC IV stages.

Sixth, we did not incorporate health-related quality-of-life data, which could be additionally implemented into the model in a future analysis. As such, long-term effectiveness was based on life expectancy instead of quality-adjusted life expectancy. Since screening results in a relatively small average gain in life expectancy, changes in quality-of-life due to psychological distress associated with the communication of screening results (e.g., of the fecal blood stool tests) or adverse events of confirmatory tests may affect the estimated incremental cost-effectiveness ratios.

Seventh, our decision model did not consider heterogeneity of the population with respect to sex or location of lesions. Only an average number of lesions were modeled and age-specific progression of adenomas was not considered.

Eighth, only index tests relevant in the Austrian setting were considered.

## Conclusions

In conclusion, based on our decision analysis and simplifying assumptions, an organized screening program with annual FIT or 10-yearly colonoscopy assuming full adherence rate is most effective. The choice between these two options may depend on the individual preferences and benefit harm-tradeoffs of screening candidates. If the first choice is 10-yearly colonoscopy, this option is cost saving and if the first choice is annual FIT, this option can be considered cost effective. The results of these analyses, including the fact boxes provided, can be used to guide decisions of payers, physicians, clinical guideline developers, and screening candidates.

## Supplementary information

**Additional file 1.** Additional information model input parameters, model calibration and sensitivity analyses. Provided input parameter data include costs, utilization of resources, survival probabilities, transition probabilities and test accuracy.

## Data Availability

All data and material are available in published, mentioned and referenced studies. Further datasets on not aggregated data analyzed during the current study are available from the corresponding author on reasonable request.

## Abbreviations

CPI: Consumer price index
CRC: Colorectal cancer
CRC-SPIN: Colorectal Cancer Simulated Population model for Incidence and Natural history
CT: Computed tomography
CTC: Computed-tomography colonography
D: Dominated
DCBE: Double-contrast barium enema
DCO: Death certificate only
DIAG: Documentation and Information System for Analysis in the Healthcare Sector (Dokumentations- und Informationssystem für Analysen im Gesundheitswesen)
Disc: Discounted
DRG: Diagnosis Related Groups
ESGE: European Society of Gastrointestinal Endoscopy
EU: European Union
EUnetHTA: European Network for Health Technology Assessment
EUR: Euro
FIT: Immunochemical fecal occult blood test
FSIG: Flexible sigmoidoscopy
gFOBT: Guaiac-based fecal occult blood test
HTA: Health technology assessment
HVB: Main Association of Austrian Social Security Institutions
ICD 10 C18: Malignant neoplasm of colon
ICD 10 C19: Malignant neoplasm of rectosigmoid junction
ICD 10 C20: Malignant neoplasm of rectum
ICD: International Classification of Diseases
ICER: Incremental cost-effectiveness ratio
IHBR: Incremental harm-benefit ratios
ISPOR: International Society for Pharmacoeconomics and Outcomes Research
LY: Life years
LYG: Life-years gained
M2-PK: M2 pyruvate kinase
MISCAN: Microsimulation Screening Analysis
MR: Magnetic resonance tomography
OECD: The Organization for Economic Cooperation and Development
SEER: Surveillance, Epidemiology, and End Results (SEER) program
SimCRC: Simulation Model of Colorectal Cancer
SMDM: Society for Medical Decision Making
UICC: Union for International Cancer Control
UMIT: University for Health Sciences, Medical Informatics and Technology
US: United States
USPSTF: United States Preventive Services Task Force
