# Supplementary material for: Effectiveness, benefit harm and cost effectiveness of colorectal cancer screening in Austria
Source: BMC Gastroenterol. 2019 Dec 5;19:209. doi: 10.1186/s12876-019-1121-y (PMC6896501; doi:10.1186/s12876-019-1121-y)
Supplement: Supplementary file 1 — Additional file 1. Additional information model input parameters, model calibration and sensitivity analyses. Provided input parameter data include costs, utilization of resources, survival probabilities, transition probabilities and test accuracy. [file 12876_2019_1121_MOESM1_ESM.pdf]

Additional file 1

Natural history parameter calibration

Calibration parameters (Table S6) were transition probabilities from adenoma to advanced adenoma, advanced adenoma to preclinical UICC stage I and from preclinical UICC stage I to stage II, III and IV as well as probabilities of being symptomatic (from any preclinical stage).

Primary calibration target was the cumulative incidence of colorectal cancer at age 75 (i.e., the risk to develop cancer by the age of 75). Secondary targets were age-specific lifetime incidence and the cancer stage distribution (detected UICC I-IV cases).

First, these target parameters were derived from an unscreened population in Austria (1995-1999) (1). Age-specific lifetime incidence was given in 5-year age groups with a peak at age 70-75. It was assumed that cancer cases reported as death certificate only (DCO cases) are severe cases and therefore, they were proportionally distributed among UICC III-IV stages. Cases with undefined cancer stages were proportionally distributed among UICC I-IV cases. Stage distribution from the US Surveillance, Epidemiology, and End Results Program (SEER) database and other modeling studies were applied for plausibility checks (2).

Age-specific adenoma incidence was derived from a calibration study of the MISCAN CRC screening model for the Netherlands (3). In this study, observed adenoma prevalence data estimated from international autopsy studies and Dutch epidemiological target data were used (3). No published Austrian data on adenoma prevalence are available.

In the second step (automated calibration), the calibration parameters were first fitted to the cumulative cancer incidence at age 75 and age-specific lifetime-risk was checked. Thereafter,

the algorithm was adapted using a weighted set of two target parameters (cumulative incidence, UICC stage distribution) as a goodness-of-fit measure.

In the third step (non-automated), marginal adjustments were performed to obtain stage distribution of UICC II-IV cancer cases. Detailed results of the calibration are reported in the Additional file 1 (see Table S1, Figure S1 and Figure S2).

Table S1. Stage distribution of incident CRC cases according to the calibrated model compared to other sources.

Stage distribution of incident CRC cases	UICC I	UICC II	UICC III	UICC IV
**Calibrated Model**	21%	25%	30%	24%
**Statistics Austria (1995-1999)**	21%	25%	30%	24%
**Statistics Austria without DCO cases**	25%	26%	26%	24%
**SEER (1975-1979)***	18%	33%	24%	25%
**MISCAN***	18%	34%	24%	25%

SEER - Surveillance, Epidemiology, and End Results Program, DCO - death certificate only, MISCAN - Microsimulation Screening Analysis, * validation

Figure S1: Age distribution of incident cases (standardized incidence).

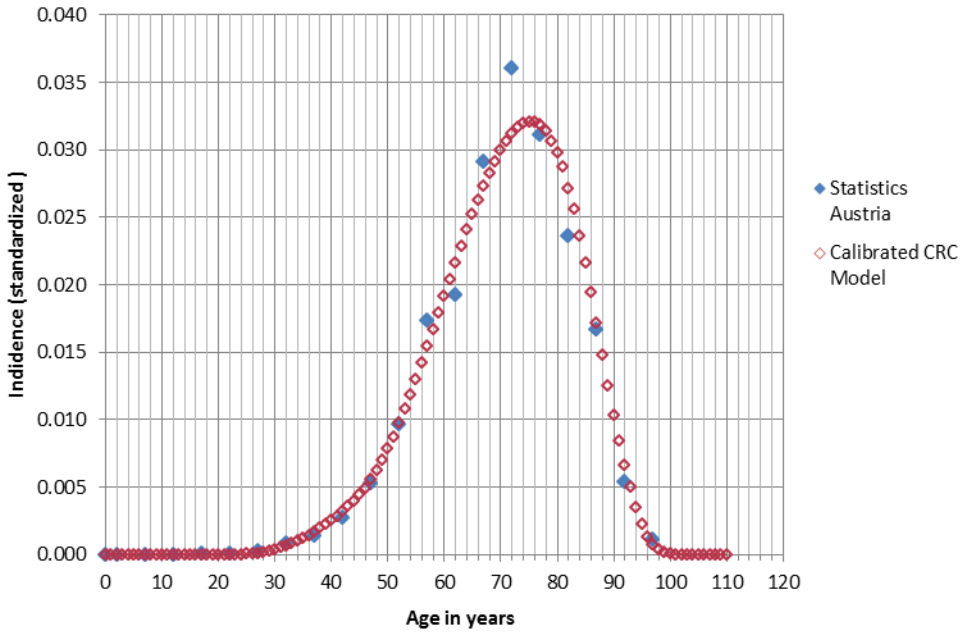

CRC – colorectal cancer

33 **Figure S2 Age distribution of incident cases (cumulative incidence).**

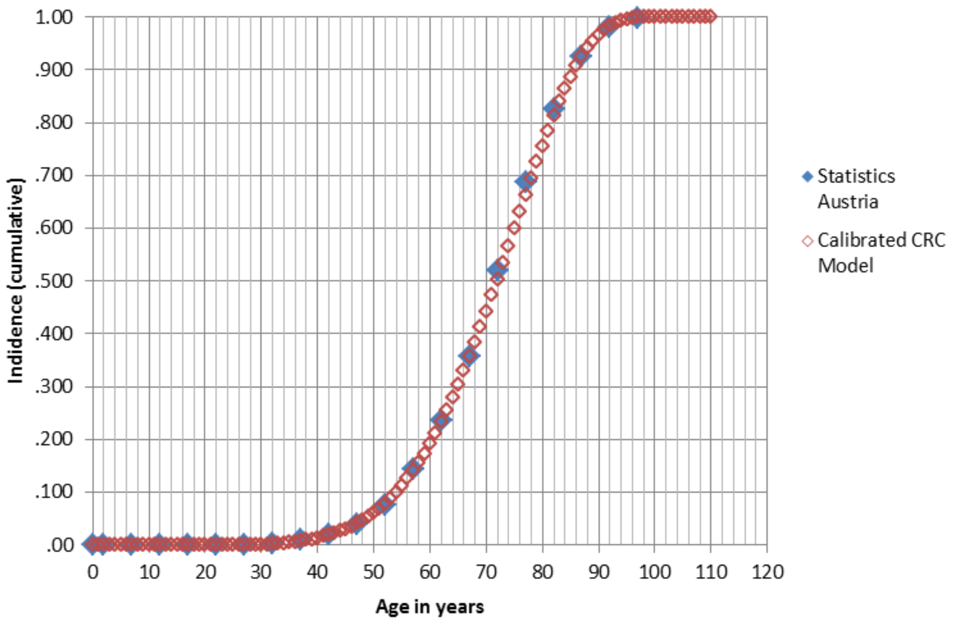

34 CRC – colorectal cancer

Test Accuracy

Table S2 Tests accuracy data of screening tests.

Test	Value	Source
**Colonoscopy**		
Sensitivity Adenomas	69.0%	Bundo et al. 2017(4)
Sensitivity Advanced adenomas	86.7%	Bundo et al. 2017(4)
Sensitivity Cancer	94.7%	Pickhardt et al. 2003(5)
Specificity	100.0%	Austrian Expert Panel
**gFOBT**		
Sensitivity Adenomas	9.5%	Zauber et al. 2008(6)
Sensitivity Advanced adenomas	23.9%	Zauber et al. 2008(6)
Sensitivity Cancer	72.2%	Hirai et al. 2016(7)
Specificity	90.0%	Hirai et al. 2016(7)
**FIT**		
Sensitivity Adenomas	7.6%	Imperiale et al. 2014(8)
Sensitivity Advanced adenomas	36.7%	Launois et al. 2014(9)
Sensitivity Cancer	87.2%	Launois et al. 2014(9)
Specificity	92.8%	Launois et al. 2014(9)

gFOBT - guaiac-fecal occult blood test, FIT - fecal immunochemical test.

Economic data

Direct-medical costs were derived from the perspective of the Austrian public health care system. Both medical outpatient- and inpatient-care costs were based on original data from the Main Association of Austrian Social Security Institutions (10). These costs are explained in more detail below taking into account the relative frequency distribution of cancer location, cancer stage and medication options, which is reported in Table S4 (1).

Costs of tests

The costs of a colonoscopy includes the cost of colonoscopy without polypectomy as well as lump compensation, outpatient visits, clinical report, the medical consultation and digital rectal examination, each as a national average of charges of internists and surgeons. The costs of polypectomy are measured as the mean costs for an endoscopic removal of polyps of the colon and the rectum.

The costs for the guaiac-based fecal occult blood test include lump compensation, outpatient visits, the medical consultation and digital rectal examination. The costs are measured as national average of charges of internists and surgeons.

The costs for the immunochemical fecal occult blood test additionally include the reagents for the examination, the laboratory examination, the transport costs and the physician's fee per patient. The costs for both types of fecal occult blood test kit are listed separately.

Staging costs

The staging costs were collected separately for colorectal cancer (i.e., cancer location ICD-10 C18 and ICD-10 C19) and rectal cancer (i.e., cancer location ICD-10 C20). These costs include in both cases the outpatient visit, laboratory work with the tumor marker (carcinoembryonic antigen), sonography of the upper abdomen and computed tomography (CT) of the abdomen and thorax. In addition, the costs for the staging of rectal cancer include also both a magnetic resonance tomography (MR) of the lesser pelvis and a rectal endosonography (10).

The staging costs were used to produce a weighted mean of these two cancer types. For this purpose, they were multiplied with the mean relative frequency of colorectal cancer and the

mean relative frequency of rectal cancer in the years 2010 to 2014 (70.31% and 29.69% respectively) (10).

Inpatient-care costs

The inpatient-care costs are provided separately for the three different cancer locations (ICD-10 C18, ICD-10 C19, ICD-10 C20) and the four cancer stages (UICC I, UICC II, UICC III and UICC IV). Thus, only patients with an identified cancer stage were considered in the available economic data. These costs were calculated using the corresponding points of the Diagnosis Related Groups (DRG) of a treatment multiplied with the estimated mean value of a DRG point (value of DRG point = EUR 1.4), separately for each UICC level and cancer location (10).

For the decision-analytic model, we aggregated this data on the four cancer stages UICC I-IV by taking into account the relative frequency of the cancer location (i.e., ICD-10 C18, ICD-10 C19 and ICD-10 C20) at each cancer stage between 2010 and 2014. Thus, we include the inpatient-care costs at UICC level as weighted mean of the three considered cancer locations.

Medication costs

The medication costs are provided for three types of medication for cancer stage UICC IV. 58.46 % of the patients receive a tyrosine kinase inhibitor (drug 1, i.e., Regorafenib (Stivarga®)) and 30.84 % of the patients an antineoplastic nucleoside analog (drug 2, i.e., Trifluridin/Tipiracil (Lonsurf®)). Additionally, 10.70 % of the patients receive Regorafenib (drug 1) plus Trifluridin (drug 2). The cost for this latter medication is calculated as the sum of the costs of the two drugs. The medication costs are further calculated for the model as weighted average of the three medication types using the above mentioned proportional shares.

Follow-up costs

The follow-up costs were provided separately for colorectal cancer (ICD-10 C18 and ICD-10 C19) and rectal cancer (ICD-10 C20). Timelines are presented in Table 10.

Follow-up costs for colorectal cancer

In the first year, the follow-up costs for colorectal cancer include a quarterly medical consultation (after 3, 6, 9 and 12 months), the tumor marker laboratory four times a year (after 3, 6, 9 and 12 months), a colonoscopy and an annual CT of the abdomen (after 12 months). In the second and the third year, the follow-up costs are similar, though no further colonoscopies are conducted.

The follow-up costs in the fourth and fifth year include a medical consultation and the measurement of tumor markers twice a year (after 42, 48, 54 and 60 months). The costs for a CT of the abdomen were further considered once in the fourth and once in fifth year (after 48 and 60 months). Moreover, the costs for a further colonoscopy were included in the fourth year (after 48 months). After the fifth year following surgery, the follow-up costs for colorectal cancer include a medical consultation and colonoscopy every five years (every 60 months).

Follow-up costs for rectal cancer

In the first year, the follow-up costs for rectal cancer include a quarterly medical consultation (after 3, 6, 9 and 12 months), the tumor marker laboratory four times a year (after 3, 6, 9 and 12 months), a rectoscopy after 6 months, a colonoscopy after 12 months and a CT of the abdomen after 6 months for 30% of the patients and after 12 months for all patients. In the second and third year, the follow-up costs are similar, though again no further colonoscopies are conducted. The rectoscopy is repeated after 24 months and for 25-30 % of the patients again after 36 months.

Similarly to the follow-up of the colorectal cancer, the follow-up costs in the fourth and fifth year include a medical consultation and measurement of tumor markers twice a year (after 42, 48, 54 and 60 months). Moreover, the abdomen was scanned with computed tomography once in the fourth and once in fifth year (after 48 and 60 months). The costs for a further colonoscopy were included in the fourth year (after 48 months). After the fifth year, the follow-up costs for colorectal cancer and rectal cancer are the same and include medical consultation and a colonoscopy every five years (i.e., every 60 months).

The overall follow-up costs in the model are calculated for each year after diagnosis as a weighted mean of colorectal cancer and rectal cancer using the relative frequency distribution (cf. staging costs).

Costs for colonoscopy screening program

The estimated annual costs for the colonoscopy screening program provided include costs for the coordinating office, management of the invitation (i.e., print and shipping of reminders), service line, evaluation, data management, quality management and public relations.

The costs for the reminders were calculated based on all people between 50 and 70 years of age in 2016. Following data provided by Statistics Austria to the HVB, these were 2369510 people in Austria on Jan 1, 2017. As the reminders for a colonoscopy screening are sent every 10 years, the costs for the invitation management are calculated for a tenth of these people, i.e., 236951 (11).

Costs for stool-based screening program

The estimated annual costs for the stool-based screening program provided include the same costs as for the colonoscopy screening, i.e., coordinating office, the management of the invitation (i.e., print and shipping of reminders), service line, evaluation, data management, quality management and public relations.

However, the costs for the management of screening invitation are higher as the reminders are sent out to all people between 40 and 75 years of age on an annual basis. Thus, the costs include screening invitations sent annually to 4016937 people in Austria (as per January 1, 2017, Statistics Austria) (11).

Costs of complications

For the calculation of the costs, only those complications of colonoscopies which result in an inpatient stay are considered. This can be further differentiated into complications followed by a surgical procedure (0.013 % of all screening colonoscopies) and complications without a surgical procedure, but still with an inpatient stay (0.03 % of all screening colonoscopies). These costs were calculated using data from the Documentation and Information System for Analyses in Healthcare (12).

End-of-life costs

End-of-life costs applied to cancer death of patients staged UICC I or UICC II include the inpatient-care costs for cancer stage UICC III and UICC IV as well as the medication costs for UICC IV. The end-of-life costs of patients staged UICC III dying from cancer include the inpatient-care costs and the medication costs for UICC IV. These end-of-life costs are calculated by taking into account the distribution of the cancer location.

Table S3 Aggregated costs of tests, staging, inpatient, medication, follow-up, screening, complications and end-of-life of colorectal and rectal cancer (Index year 2017) - Extended.

Item	Costs at index year 2017, EUR
**Costs for tests**	
Colonoscopy	228.21
Polypectomy	63.97
gFOBT	36.52
iFOBT	41.11
gFOBT (stool test only)	0.83
iFOBT (stool test only)	0.89
**Staging costs**	
Colorectal cancer	404.38
Rectal cancer	595.86
**Aggregated staging costs**	461.22
**Inpatient-care costs (cancer location-cancer stage)**	
ICD-10 C18 UICC I	14094.73
ICD-10 C18 UICC II	19665.00
ICD-10 C18 UICC III	19342.20
ICD-10 C18 UICC IV	24069.73
ICD-10 C19 UICC I	13839.35

**Item**	**Costs at index year 2017, EUR**
ICD-10 C19 UICC II	13745.38
ICD-10 C19 UICC III	14897.63
ICD-10 C19 UICC IV	18379.95
ICD-10 C20 UICC I	13353.12
ICD-10 C20 UICC II	16699.57
ICD-10 C20 UICC III	19057.20
ICD-10 C20 UICC IV	24867.53
**Aggregated inpatient costs UICC I**	**13830.58**
**Aggregated inpatient costs UICC II**	**18699.11**
**Aggregated inpatient costs UICC III**	**19037.65**
**Aggregated inpatient costs UICC IV**	**24059.44**
**Medication costs (UICC IV)**	
Regorafenib (Stivarga®)	12373.50
Trifluridin/Tipiracil (Lonsurf®)	9327.70
Stivarga® plus Lonsurf®	21701.20
**Aggregated medication costs**	**12433.00**
**Follow-up costs**	
Year 1 (colorectal cancer)	531.05
Year 2 (colorectal cancer)	346.01
Year 3 (colorectal cancer)	346.01
Year 4 (colorectal cancer)	418.95
Year 5 (colorectal cancer)	233.91
Year 9, year 14, lifelong every 60 months (colorectal cancer)	228.21
Year 1 (rectal cancer).	600.82
Year 2 (rectal cancer)	415.68
Year 3 (rectal cancer)	355.09
Year 4 (rectal cancer)	418.95
Year 5 (rectal cancer)	242.99

Item	Costs at index year 2017, EUR
Year 9, year 14, lifelong every 60 months (rectal cancer)	228.21
**Aggregated follow-up costs: year 1**	**551.76**
**Aggregated follow-up costs: year 2**	**366.69**
**Aggregated follow-up costs: year 3**	**348.71**
**Aggregated follow-up costs: year 4**	**418.95**
**Aggregated follow-up costs: year 5**	**236.61**
**Aggregated follow-up costs: year 9, 14, lifelong every 60 months**	**228.21**
**Costs for screening program**	
Costs for colonoscopy screening program	1950353.17
Costs for stool-based screening program	4118142.33
**Costs of complications**	
Surgical procedures	23258.11
Inpatient stay	5250.33
**End-of-life costs**	
One-time costs, cancer death at UICC I and UICC II	55530.09
One-time costs, cancer death at UICC III	36492.45

gFOBT - guaiac-fecal occult blood test, FIT - fecal immunochemical test, EUR - Euro, ICD-10 C18 - malignant neoplasm of colon, ICD-10 C19 - malignant neoplasm of rectosigmoid junction, ICD-10 C20 - malignant neoplasm of rectum, UICC - Union for International Cancer Control classification.

Table S4 Relative frequency distribution of cancer stage, cancer location, and medication options used for cost calculations.

Item	Relative frequency	Source
**Cancer location per cancer stage***		
UICC I		
ICD-10 C18	0.6182	Statistics Austria (1)
ICD-10 C19	0.0391	Statistics Austria (1)
ICD-10 C20	0.3427	Statistics Austria (1)
UICC II		
ICD-10 C18	0.7109	Statistics Austria (1)
ICD-10 C19	0.0368	Statistics Austria (1)
ICD-10 C20	0.2524	Statistics Austria (1)
UICC III		
ICD-10 C18	0.6400	Statistics Austria (1)
ICD-10 C19	0.0486	Statistics Austria (1)
ICD-10 C20	0.3115	Statistics Austria (1)
UICC IV		
ICD-10 C18	0.6902	Statistics Austria (1)
ICD-10 C19	0.0397	Statistics Austria (1)
ICD-10 C20	0.2701	Statistics Austria (1)
**Cancer location**		
Colorectal cancer (ICD-10 C18 + ICD-10 C19)	0.7032	Statistics Austria (1)
Rectal cancer (ICD-10 C20)	0.2968	Statistics Austria (1)
**Medication options****		
Option 1 - Regorafenib (Stivarga®)	0.5846	HVB (10)
Option 2 - Trifluridin (Lonsurf®)	0.3084	HVB (10)
Option 3 - Regorafenib + Trifluridin	0.1071	HVB (10)

*mean values for the years 2010-2014, ** values for the year 2016, HBV - Main Association of Austrian Social Security Institutions, ICD-10 C18 - malignant neoplasm of colon, ICD-10 C19 - malignant neoplasm of rectosigmoid junction, ICD-10 C20 malignant neoplasm of rectum, UICC - Union for International Cancer Control classification.

Table S5. Timeline of follow-up cost elements for colorectal and rectal cancer.

Year	Follow-up cost elements	Months after surgery
**Colorectal and rectal cancer**		
1, 2, 3, 4, 5, 9, 14, lifelong every 5 years	Medical consultation	3, 6, 9, 12, 15, 18, 21, 24, 27, 30, 33, 36<br>42, 48, 54, 60<br>108, 168, 228, ...
1, 2, 3, 4, 5	Tumor marker laboratory (“Tumormarker-Labor”)	3, 6, 9, 12, 15, 18, 21, 24, 27, 30, 33, 36<br>42, 48, 54, 60
1, 4, 9, 14, lifelong every 5 years	Colonoscopy	12, 48,<br>108, 168, 228, …
**Colorectal cancer**		
1, 2, 3	CT of the abdomen	12, 24, 36, 48, 60
**Rectal cancer**		
1, 2, 3, 5	Rectoscopy	6, 24, 36 (25-30%)<br>60 (25-30%)
1, 2, 3, 4, 5	CT of the abdomen	6 (30%), 12, 18 (30%), 24, 36, 48, 60

CT - computed tomography.

Further model parameters

Table S6. Natural history model parameters and screening adverse effects

Transition From	To	Age (years)	Annual probability (annual rate)	Source
No lesion	Adenoma	0-19	0.00200*	
		20-29	0.00400*	
		30-39	0.00600*	
		40-44	0.02400*	
		45-49	0.02900*	
		50-54	0.03000*	
		55-59	0.03400*	Goede et al. 2013 (3)
		60-64	0.04100*	
		65-69	0.04700*	
		70-74	0.05700*	
		75-79	0.03800*	
		80-84	0.03600*	
		85-120	0.01000*	
Adenoma	Advanced adenoma		0.016273	calibrated
Advanced adenoma	UICC I undetected		0.027150	calibrated
UICC I undetected	UICC II undetected		0.500000	calibrated
UICC II undetected	UICC III undetected		0.600000	calibrated
UICC III undetected	UICC IV undetected		0.700000	calibrated
UICC I undetected	UICC I detected by symptoms		0.105000	calibrated
UICC II undetected	UICC II detected by symptoms		0.205000	calibrated
UICC III undetected	UICC III detected by symptoms		0.450000	calibrated
UICC IV undetected	UICC IV detected by symptoms		1.000000	calibrated
Screening adverse effects				
Death from colonoscopy			0.002900	Reumkens et al. 2016 (13)
Hospitalization			0.000420	Austrian Colonoscopy Registry (14)

*calibrated to autopsy studies.
Calibrated - to cumulative and age-specific incidence of colorectal cancer and UICC stage distribution of incident cases in Austria - Statistics Austria 1995-1999(1), UICC - Union for International Cancer Control classification.

Table S7. Relative survival probability for symptomatic-detected colorectal cancer patients.

Year post first diagnosis	Relative survival probability for symptomatic-detected colorectal cancer patients with first CRC diagnosis			
	**UICC I**	**UICC II**	**UICC III**	**UICC IV**
1-year	0.915	0.892	0.851	0.470
2-year	0.980	0.961	0.888	0.615
3-year	0.983	0.967	0.905	0.645
4-year	0.978	0.964	0.911	0.721
5-year	0.991	0.966	0.939	0.806
6-year	0.993	0.972	0.950	0.840
7-year	0.994	0.977	0.959	0.869
8-year	0.995	0.981	0.966	0.896
9-year	0.996	0.985	0.973	0.920
10-year	0.997	0.989	0.980	0.942
11-year	0.998	0.992	0.985	0.963
12-year	0.999	0.995	0.991	0.982
13-year	1.000	0.997	0.995	1.000
14-year	1.000	1.000	1.000	1.000

Recalculated based on averaged relative survival probabilities from Statistics Austria 2010-2014 for first diagnosis (ICD 10 C18 - malignant neoplasm of colon, ICD 10 C19 - malignant neoplasm of rectosigmoid junction, ICD 10 C20 - malignant neoplasm of rectum) including screen and non-screen detected patients.
CRC - colorectal cancer, UICC - Union for International Cancer Control classification.

Table S8. Relative survival probability for screen-detected colorectal cancer patients.

Year post first diagnosis	Relative survival probability for screen-detected colorectal cancer patients with first CRC diagnosis			
	UICC I	UICC II	UICC III	UICC IV
1-year	0.975	0.967	0.948	0.675
2-year	0.994	0.989	0.961	0.777
3-year	0.995	0.990	0.968	0.796
4-year	0.994	0.989	0.970	0.843
5-year	0.997	0.990	0.979	0.894
6-year	0.998	0.992	0.983	0.913
7-year	0.998	0.993	0.986	0.930
8-year	0.999	0.994	0.989	0.944
9-year	0.999	0.996	0.991	0.958
10-year	0.999	0.997	0.993	0.970
11-year	1.000	0.998	0.995	0.981
12-year	1.000	0.998	0.997	0.991
13-year	1.000	0.999	0.999	1.000
14-year	1.000	1.000	1.000	1.000

Recalculated based on averaged relative survival probabilities from Statistics Austria 2010-2014 for first diagnosis (ICD 10 C18 - malignant neoplasm of colon, ICD 10 C19 - malignant neoplasm of rectosigmoid junction, ICD 10 C20 - malignant neoplasm of rectum) including screen and non-screen detected patients.

CRC - colorectal cancer, UICC - Union for International Cancer Control classification.

Table S9. Relative survival probability for patients diagnosed with colorectal cancer (screen and symptomatic detected).

Year post first diagnosis	Relative survival probability for patients with first CRC diagnosis			
	**UICC I**	**UICC II**	**UICC III**	**UICC IV**
1-year	92.9	91.0	87.4	51.8
2-year	98.3	96.8	90.5	65.3
3-year	98.6	97.2	92.0	68.0
4-year	98.2	97.0	92.5	75.0
5-year	99.2	97.1	94.9	82.7
6-year	99.4	97.6	95.8	86.0
7-year	99.5	98.1	96.6	88.8
8-year	99.6	98.4	97.2	91.2
9-year	99.7	98.8	97.8	93.3
10-year	99.8	99.1	98.3	95.2
11-year	99.9	99.3	98.8	97.0
12-year	99.9	99.6	99.2	98.5
13-year	100.0	99.8	99.6	100.0
14-year	100.0	100.0	100.0	100.0

Averaged data from Statistics Austria 2010-2014 for diagnosis (ICD-10 C18-C20) including screen and non-screen detected patients for 1-year-to 5-year, 6-year and following data were extrapolated applying logarithmic functions to mortality probabilities.
CRC - colorectal cancer, UICC - Union for International Cancer Control classification.

Base-case analysis screening-related benefits and harms

Table S10 Outcome of screening programs to prevent colorectal cancer.

Outcome	Screening strategy:	10-yearly colonoscopy	Annual gFOBT	Annual FIT
**Life-years gained**		394	480	491
**CRC-related deaths averted**		31	35	35
**CRC cases averted**		62	66	69
**Additional complications due to colonoscopy (hospital admissions)**		1.17	1.49	1.23
**Total positive test results**		679	2797	2206

Numbers pertain to a cohort of 1000 persons 40 years of age who were followed until death in comparison to No Screening, CRC -colorectal cancer, gFOBT - guaiac-fecal occult blood test screening strategy, FIT - fecal immunochemical test screening strategy. FIT and gFOBT: 40-75 years old average - risk men and women. Colonoscopy: 50-70 years old average - risk men and women, all screening strategies include index testing, further diagnostics (including colonoscopy), surveillance (colonoscopy), treatment and follow up interventions.

Sensitivity analyses test accuracy

Figure S3 Sensitivity analysis on test accuracy for gFOBT and FIT impact on life years.

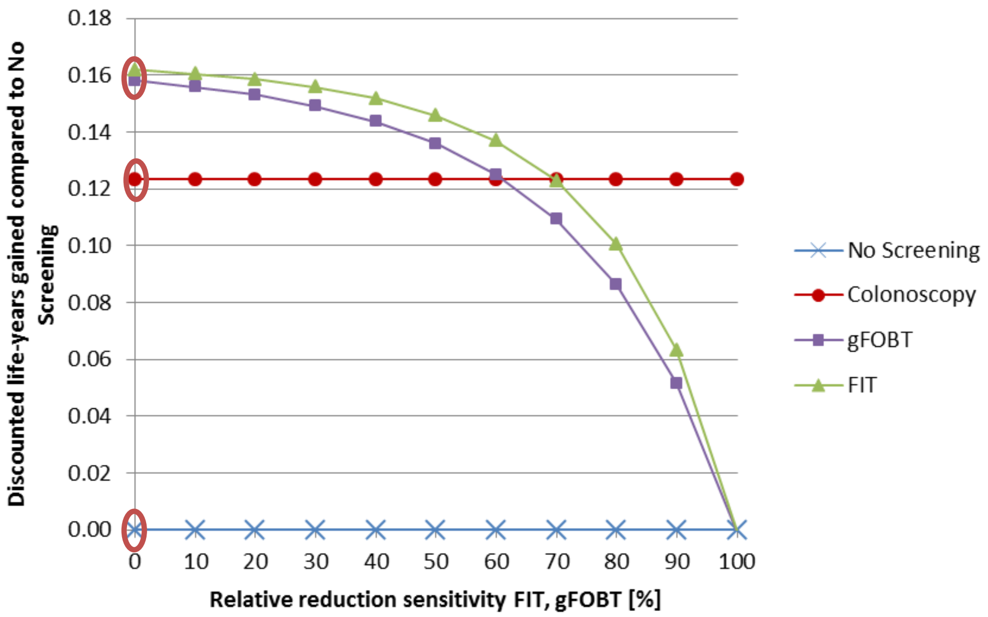

gFOBT - guaiac-fecal occult blood test strategy, FIT - fecal immunochemical test strategy. FIT and gFOBT: 40-75 years old average - risk men and women, annual. Colonoscopy: 50-70 years old average - risk men and women, 10-yearly, all screening strategies include index testing, further diagnostics (including colonoscopy), surveillance (colonoscopy), treatment and follow up interventions.
Red circles represent base case.

Figure S4 Sensitivity analysis on test accuracy for gFOBT and FIT impact on ICER.

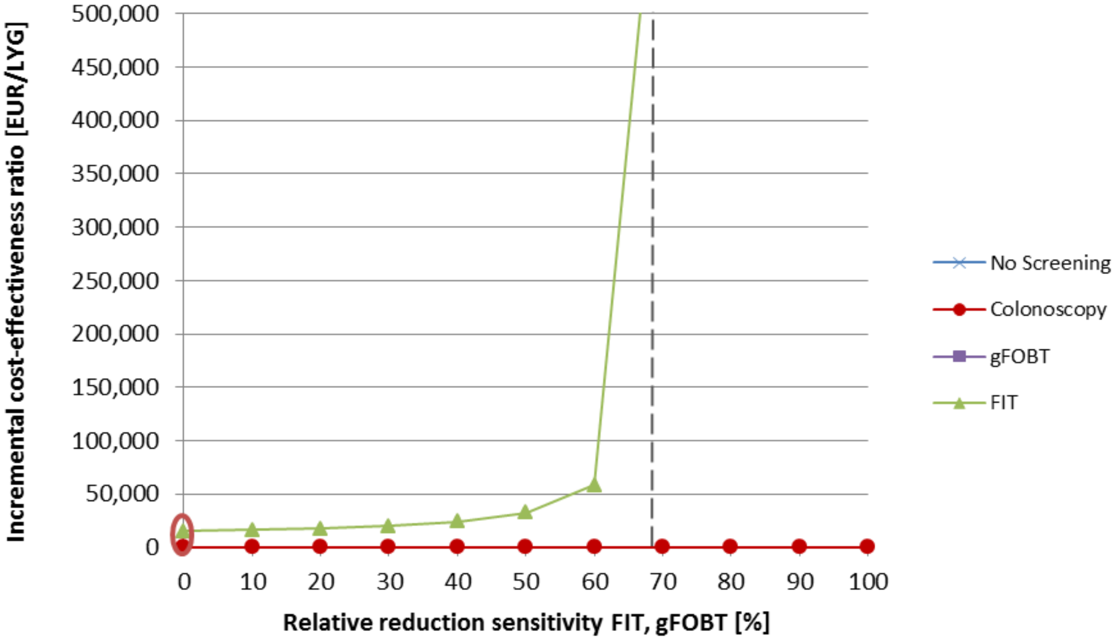

gFOBT - guaiac-fecal occult blood test strategy, FIT - fecal immunochemical test strategy, EUR - Euro, LYG - life-years gained. FIT and gFOBT: 40-75 years old average - risk men and women, annual. Colonoscopy: 50-70 years old average - risk men and women, 10-yearly, all screening strategies include index testing, further diagnostics (including colonoscopy), surveillance (colonoscopy), treatment and follow up interventions. Red circle represents base case. gFOBT and No Screening are dominated strategies. Therefore, they are not shown in the graphic.

Table S11 Calculative 10-year sensitivity of fecal blood tests in comparison to sensitivity of colonoscopy.

	Sensitivity FIT	10-year sensitivity* FIT	Sensitivity gFOBT	10-year sensitivity* gFOBT	Sensitivity colonoscopy
Adenoma	0.076	0.546	0.095	0.631	0.690
Advanced adenoma	0.367	0.990	0.239	0.935	0.867
Cancer	0.872	1.000	0.647	1.000	0.947

*assuming independent test sensitivities for repeated screening tests.
gFOBT - guaiac-fecal occult blood test strategy, FIT fecal immunochemical test strategy, FIT and gFOBT: 40-75 years old average - risk men and women. Colonoscopy: 50-70 years old average - risk men and women, all screening strategies include index testing, further diagnostics (including colonoscopy), surveillance (colonoscopy), treatment and follow up interventions.

Two-way sensitivity analyses

Figure 7, Figure 8 and Figure 9 show the results of the two-way sensitivity analyses when the sensitivity of fecal occult blood tests (gFOBT, FIT) and the sensitivity of colonoscopy are varied simultaneously assuming a willingness-to-pay thresholds of EUR 10000/LYG, EUR 20000/LYG and EUR 30000/LYG, respectively. The graphics read as follows: the sensitivity parameters for the fecal occult blood tests are reduced by up to 50% and increased by up to 10% (x-axis). The same variation is assumed for the sensitivity of colonoscopy displayed on the y-axis. Depending on the combinations of these two parameters on the x- and y-axis, the shade of the area defines the cost-effective screening strategy given the respective willingness-to-pay threshold. The combination of the factor 1 on the x-axis (sensitivity fecal occult blood tests) and 1 on the y-axis (sensitivity colonoscopy) displays the base-case results. Assuming a willingness-to-pay threshold of EUR 20000/LYG (Figure 8), FIT is cost-effective for the base-case parameter set. Assuming a 20% reduction in the sensitivity of the fecal occult blood tests and a 10% increase in the sensitivity of colonoscopy would lead to 10-yearly colonoscopy screening being the cost-effective strategy.

Figure S5 Two-way sensitivity analysis of test sensitivities with a WTP of EUR 10,000 LYG.

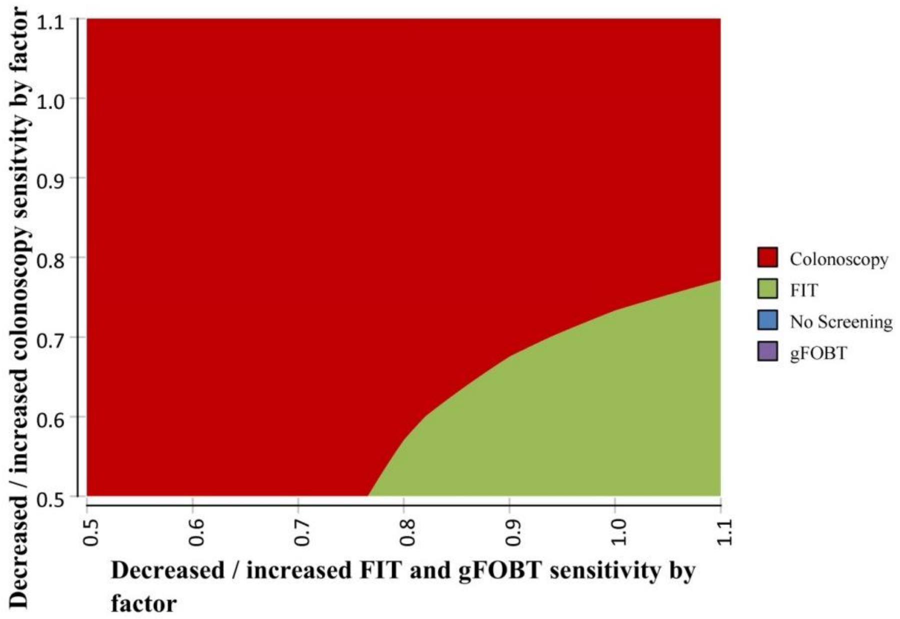

gFOBT - guaiac-fecal occult blood test screening strategy, FIT - fecal immunochemical test screening strategy, WTP willingness-to-pay, EUR - Euro, LYG - life-years gained.

Figure S6 Two-way sensitivity analysis of test sensitivities with a WTP of EUR 20,000 LYG.

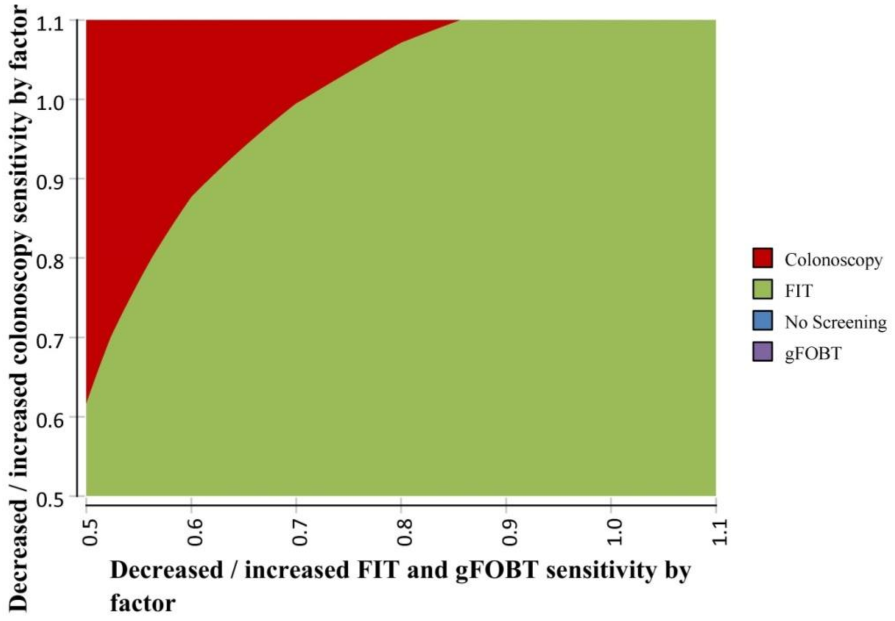

gFOBT - guaiac-fecal occult blood test screening strategy, FIT - fecal immunochemical test screening strategy, WTP willingness-to-pay, EUR - Euro, LYG - life-years gained.

Figure SS7 Two-way sensitivity analysis of test sensitivities with a WTP of EUR 30,000 LYG.

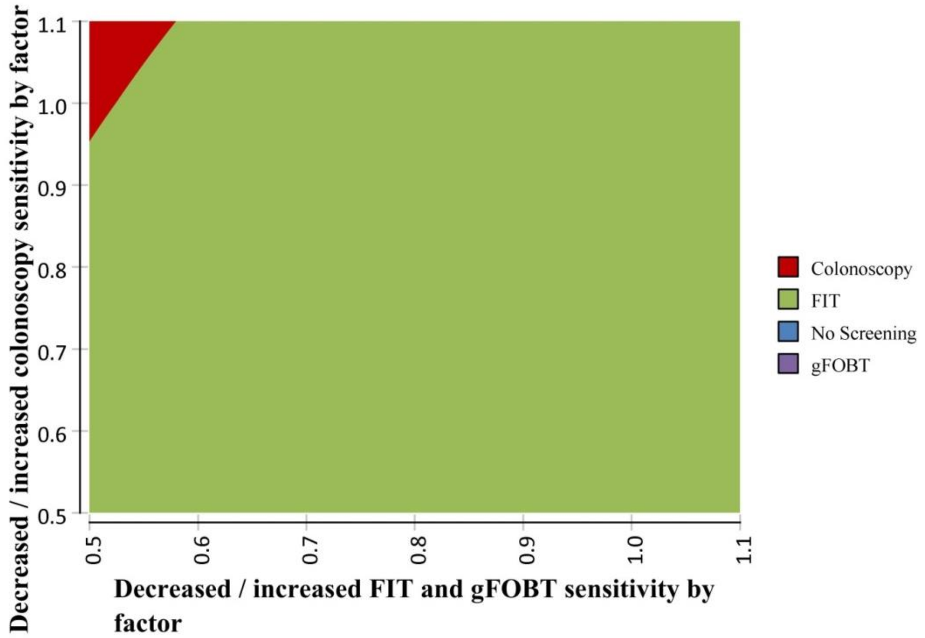

gFOBT - guaiac-fecal occult blood test screening strategy, FIT - fecal immunochemical test screening strategy, WTP willingness-to-pay, EUR - Euro, LYG - life-years gained.

Figure 10, Figure 11 and Figure 12 show the results of the two-way sensitivity analyses when the participation rates of fecal occult blood tests (gFOBT, FIT) and colonoscopy are varied simultaneously assuming willingness-to-pay thresholds of EUR 10000/LYG, EUR 20000/LYG and EUR 30000/LYG, respectively. The participation rates for the both fecal occult blood tests are assumed to be equal. The graphics read as follows: the participation rates are assumed to vary between 10% and 100%. With increasing willingness-to-pay thresholds 10-yearly colonoscopy screening (symbolized by a red shaded area for the combinations of participation rates) would be the preferred option only for high participation rates of colonoscopy screening and low participation rates of the annual FIT screening.

Figure S8. Two-way sensitivity analysis of test participation rates with a WTP of EUR 10,000 LYG.

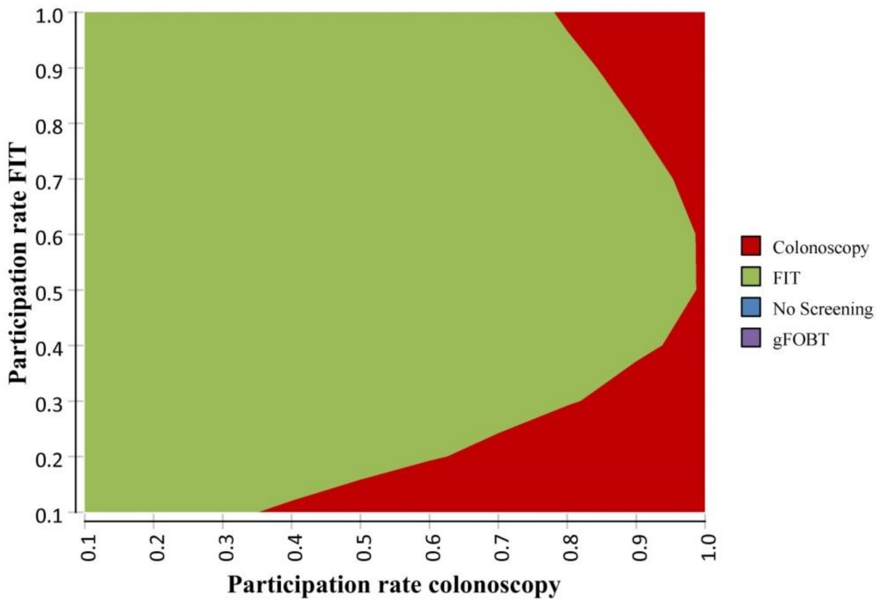

gFOBT - guaiac-fecal occult blood test screening strategy, FIT - fecal immunochemical test screening strategy, WTP willingness-to-pay, EUR - Euro, LYG - life-years gained.

Figure S9. Two-way sensitivity analysis of test participation rates with a WTP of EUR 20,000 LYG.

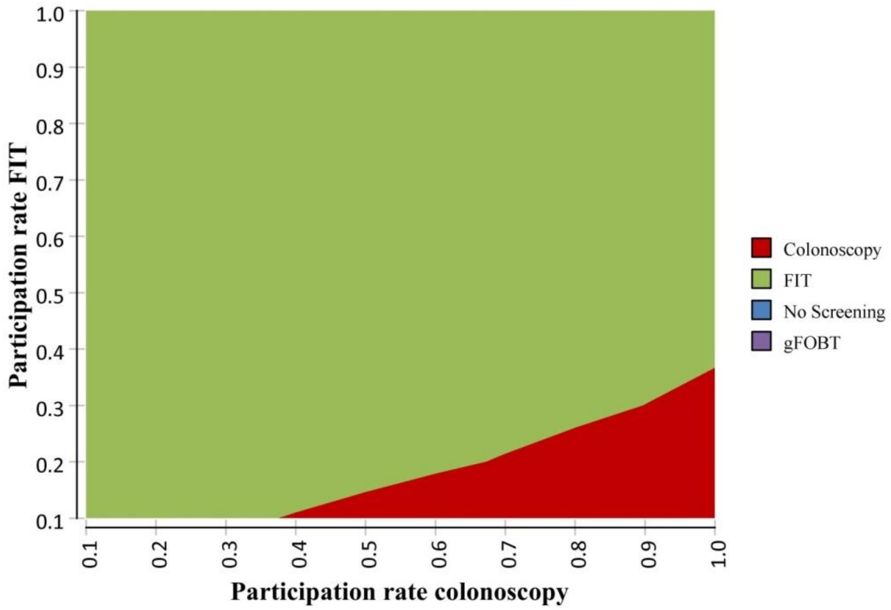

gFOBT - guaiac-fecal occult blood test screening strategy, FIT - fecal immunochemical test screening strategy, WTP willingness-to-pay, EUR - Euro, LYG - life-years gained.

Figure S10. Two-way sensitivity analysis of test participation rates with a WTP of EUR 30,000 LYG.

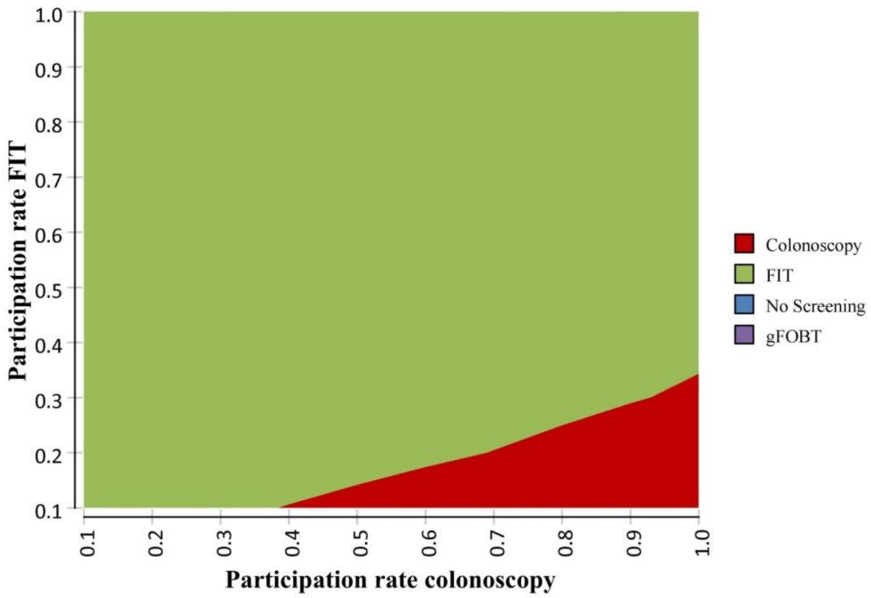

gFOBT - guaiac-fecal occult blood test screening strategy, FIT - fecal immunochemical test screening strategy, WTP willingness-to-pay, EUR - Euro, LYG - life-years gained.

References

1. Statistics Austria. Personal communication. 2017.

2. Zauber A, Knudsen A, Rutter C, Lansdorp-Vogelaar I, Kuntz K. Evaluating the benefits and harms of colorectal cancer screening strategies: a collaborative modeling approach. Available from: https://www.uspreventiveservicestaskforce.org/Home/GetFile/1/16540/cisnet-draft-modeling-report/pdf [Accessed on December 27, 2017]. AHRQ Publication. 2015(14-05203).

3. Goede SL, van Roon AHC, Reijerink JCIY, van Vuuren AJ, Lansdorp-Vogelaar I, Habbema JDF, et al. Cost-effectiveness of one versus two sample faecal immunochemical testing for colorectal cancer screening. Gut. 2013;62(5):727-34.

4. Bundo M, Jahn B, Arvandi M, Sroczynski G, Siebert U, editors. Adenoma Miss Rate of Conventional Colonoscopy: a Systematic Review and Meta-Analysis [forthcoming poster presentation] EbM-Kongress 2018; 2018; Graz, Austria.

5. Pickhardt PJ, Hassan C, Halligan S, Marmo R. Colorectal cancer: CT colonography and colonoscopy for detection--systematic review and meta-analysis. Radiology. 2011;259(2):393-405.

6. Zauber AG, Lansdorp-Vogelaar I, Knudsen AB, Wilschut J, van Ballegooijen M, Kuntz KM. Evaluating test strategies for colorectal cancer screening: a decision analysis for the U.S. Preventive Services Task Force. Annals of internal medicine. 2008.

7. Hirai HW, Tsoi KK, Chan JY, Wong SH, Ching JY, Wong MC, et al. Systematic review with meta-analysis: faecal occult blood tests show lower colorectal cancer detection rates in the proximal colon in colonoscopy-verified diagnostic studies. Alimentary pharmacology & therapeutics. 2016;43(7):755-64.

8. Imperiale TF, Ransohoff DF, Itzkowitz SH. Multitarget stool DNA testing for colorectal-cancer screening. The New England journal of medicine. 2014;371(2):187-8.

9. Launois R, Le Moine JG, Uzzan B, Fiestas Navarrete LI, Benamouzig R. Systematic review and bivariate/HSROC random-effect meta-analysis of immunochemical and guaiac-based fecal occult blood tests for colorectal cancer screening. European journal of gastroenterology & hepatology. 2014;26(9):978-89.

10. Main Association of Austrian Security Institutions (Hauptverband der österreichischen Sozialversicherungsträger-HBV). Original data on medical costs. Personal communication. 2017.

11. Statistics Austria. Bevölkerung nach Alter und Geschlecht. Available from; http://www.statistik.at/web_de/statistiken/menschen_und_gesellschaft/bevoelkerung/bevoelkerungsstruktur/bevoelkerung_nach_alter_geschlecht/index.html [Accessed on December 27, 2017].

12. Main Association of Austrian Security Institutions (Hauptverband der österreichischen Sozialversicherungsträger-HBV). Personal communication. 2017.

13. Reumkens A, Rondagh EJ, Bakker CM, Winkens B, Masclee AA, Sanduleanu S. Post-Colonoscopy Complications: A Systematic Review, Time Trends, and Meta-Analysis of Population-Based Studies. The American journal of gastroenterology. 2016;111(8):1092-101.

14. Austrian Colonoscopy Registry. Personal communication. 2017.
